# The Quality in Acute Stroke Care (QASC) global scale-up using a cascading facilitation framework: a qualitative process evaluation

**DOI:** 10.1186/s12913-024-10617-9

**Published:** 2024-01-29

**Authors:** Elizabeth McInnes, Simeon Dale, Kathleen Bagot, Kelly Coughlan, Jeremy Grimshaw, Waltraud Pfeilschifter, Dominique A. Cadilhac, Thomas Fischer, Jan van der Merwe, QASC Europe Steering Committee, QASC Europe Implementation Committee, Sandy Middleton

**Affiliations:** 1 Nursing Research Institute, St Vincent’s Health Network Sydney, St Vincent’s Hospital Melbourne; and Australian Catholic University, Sydney, Australia; 2 School of Nursing, Midwifery and Paramedicine, Australian Catholic University, Sydney, Australia; 3 Centre for Practice-Changing Research (CPCR), Ottawa Health Research Institute, Ottawa Hospital - General Campus; and University of Ottawa, Ottawa, ON Canada; 4 Department of Neurology and Clinical Neurophysiology, Städtisches Klinikum Lüneburg, Lüneburg, Germany; 5 Department of Neurology, Germany Centre of Neurology and Neurosurgery, Goethe University, Frankfurt am Main, University Hospital Frankfurt, Frankfurt, Germany; 6 Translational Public Health Division, Stroke and Ageing Research, School of Clinical Sciences, Monash University, Melbourne, Australia; 7 Public Health, Stroke Division, The Florey Institute of Neuroscience and Mental Health, University of Melbourne, Melbourne, Australia; 8 Angels Initiative, Ingelheim, Germany

**Keywords:** Implementation science, Facilitation, Stroke, Clinical protocols, Nursing research, Quality improvement, Learning health system.

## Abstract

**Background:**

Variation in hospital stroke care is problematic. The Quality in Acute Stroke (QASC) Australia trial demonstrated reductions in death and disability through supported implementation of nurse-led, evidence-based protocols to manage fever, hyperglycaemia (sugar) and swallowing (FeSS Protocols) following stroke. Subsequently, a pre-test/post-test study was conducted in acute stroke wards in 64 hospitals in 17 European countries to evaluate upscale of the FeSS Protocols. Implementation across countries was underpinned by a cascading facilitation framework of multi-stakeholder support involving academic partners and a not-for-profit health organisation, the Angels Initiative (the industry partner), that operates to promote evidence-based treatments in stroke centres. .We report here an *a priori* qualitative process evaluation undertaken to identify factors that influenced international implementation of the FeSS Protocols using a cascading facilitation framework.

**Methods:**

The sampling frame for interviews was: (1) Executives/Steering Committee members, consisting of academics, the Angels Initiative and senior project team, (2) Angel Team leaders (managers of Angel Consultants), (3) Angel Consultants (responsible for assisting facilitation of FeSS Protocols into multiple hospitals) and (4) Country Co-ordinators (senior stroke nurses with country and hospital-level responsibilities for facilitating the introduction of the FeSS Protocols). A semi-structured interview elicited participant views on the factorsthat influenced engagement of stakeholders with the project and preparation for and implementation of the FeSS Protocol upscale. Interviews were recorded, transcribed verbatim and analysed inductively within NVivo.

**Results:**

Individual (*n* = 13) and three group interviews (3 participants in each group) were undertaken. Three main themes with sub-themes were identified that represented key factors influencing upscale: (1) *readiness for change (sub-themes: negotiating expectations; intervention feasible and acceptable; shared goal of evidence-based stroke management); (2) roles and relationships (sub-themes: defining and establishing roles; harnessing nurse champions) and (3) managing multiple changes (sub-themes: accommodating and responding to variation; more than clinical change; multi-layered communication framework)*.

**Conclusion:**

A cascading facilitation model involving a partnership between evidence producers (academic partners), knowledge brokers (industry partner, Angels Initiative) and evidence adopters (stroke clinicians) overcame multiple challenges involved in international evidence translation. Capacity to manage, negotiate and adapt to multi-level changes and strategic engagement of different stakeholders supported adoption of nurse-initiated stroke protocols within Europe. This model has promise for other large-scale evidence translation programs.

**Supplementary Information:**

The online version contains supplementary material available at 10.1186/s12913-024-10617-9.

## What is already known

- Evidence from a body of research including a randomised controlled trial demonstrates that implementation of nurse-led protocols to manage fever, hyperglycaemia (sugar) and swallowing following stroke significantly improves stroke care and patient outcomes.
- Worldwide, strategies are needed to increase the uptake of evidence-based stroke care to address variability in-hospital acute stroke care.
- There is little research on strategies for international upscale of evidence-based care that involves collaboration between researchers, non-government organisations, health services and not for profit organisations.

## What this paper adds

- A unique model of multi-stakeholder support, involving researchers, health services and a not-for profit organisation, called ‘cascading facilitation,’ enabled global scale-up of evidence-based acute stroke protocols across 17 European countries.
- Evidence-based clinical change within hospitals, can be initiated and facilitated outside of the healthcare system, through a university-industry collaboration, where there is a shared goal of optimal care, clear roles and a multi-layered communication system.
- Cascading facilitation could be used for other global implementation evidence translation initiatives.

## Introduction

 Stroke remains the second leading cause of death and third leading cause of death and disability combined worldwide in 2019 [1]. Uptake of evidence-based clinical guidelines improves patient outcomes and survival after stroke [2–5]. Considerable variability in the delivery of evidence-based acute stroke care management [1] indicates that strategies are needed to maximise uptake of evidence-based care to meet targets for the Action Plan for Stroke in Europe [6]. 

Implementing change and the uptake of practice guidelines for clinical care is difficult [7, 8]. Collaborations between clinicians, researchers, non-government organisations, health services and not for profit organisations have been reported as effective strategies for knowledge translation [8]. These collaborations are referred to as Academic-Industry Partnerships, University-Industry collaborations or Public-Private Partnerships [9]. University-Industry collaborations are well established within management or technology fields [9–12]. Collaborative partners bring different perspectives; the focus of university academics may differ to those in industry who have on-ground knowledge of health systems [12–14]. Understanding how these collaborations work in a health care context to enable uptake of clinical guidelines across countries is valuable for informing future upscale efforts.

The QASC Europe pre-test/post-test study [15] aimed to upscale the fever, hyperglycaemia (sugar) and swallowing (FeSS) protocols in hospitals in Europe through a university-industry collaboration that involved a lead University research institute (i.e. the Nursing Research Institute) and the Angels Initiative (the industry partner). The lead University partner researchers led the seminal Quality in Acute Stroke Care (QASC) cluster randomised controlled trial conducted in the Australian state of New South Wales (NSW) and subsequent NSW-wide scale-up of the implementation of the Fever, Sugar, Swallowing (FeSS) Protocols, for patients in acute stroke units [2, 16, 17]. This body of research showed that facilitated implementation of these nurse-led evidence-based protocols for managing fever, hyperglycaemia, and swallowing dysfunction reduced death and dependency 90-days post-discharge [2] and increased longer-term survival (median of four years) [3]. A subsequent study that aimed to evaluate the cost-effectiveness of implementing the FeSS protocols across Australia, demonstrated that if the protocols were implemented over a five-year period, more than 1,500 deaths would be prevented and there would be savings of AUD 65 million [18]. Following this research, the Co-founders and Project Leads of the Angels Initiative contacted the QASC Trial lead investigator (SM) about a potential collaboration to implement and evaluate upscale of the FeSS Protocols across Europe.

The Angels Initiative, (the Acute Networks strivinG for ExceLlence in Stroke) Initiative is a not-for-profit healthcare program established in 2015 ‘to increase the number of patients treated in stroke ready hospitals and to optimise the quality of treatment in all existing stroke centres’ [19]. Working in partnership with the European Stroke Organisation (ESO), the World Stroke Organisation (WSO), the Stroke Alliance for Europe (SAFE), and other national stroke societies and health institutions, the Angels Initiative has over 7,500 registered hospitals in 140 countries. Angel Consultant facilitators (who have a health science or biomedical background), are managed by Angel Team Leaders, work with doctors, nurses and ambulance crews to translate evidence on stroke treatment and diagnosis within a structured program of training, change management and education. Prior to the QASC Europe Project, the focus of the Angels Initiative was to improve hyper-acute stroke care, primarily working with emergency department medical practitioners.

Although university-industry collaborations are strongly encouraged by national and international health research funding bodies [20] it is important to understand the factors that influence the success and value of these novel partnerships in facilitating international implementation and uptake of clinical protocols. We conducted a qualitative process evaluation of the international scale-up of the FeSS Protocols to 64 European hospitals in 17 countries. The rationale was to identify, from the perspective of a range of stakeholders involved in the projects, the factors that promoted upscale of the protocols using a university-industry partnership as a vehicle for evidence translation.

## Methods

A qualitative descriptive design using individual and group interviews conducted with representatives of the university-industry collaboration from Europe and Australia, and stakeholders from multiple European countries and hospitals. The process evaluation was conducted prior to the end of the pre-test/post-test study conducted in Europe and the results were not known at the time of interviews. The reporting of this study followed the COREQ (COnsolidated criteria for REporting Qualitative research) guidelines. A separate pre-test/post-test study evaluated the intervention impact on processes of care [15]. 

### Cascading facilitation

**Fig. 1:**
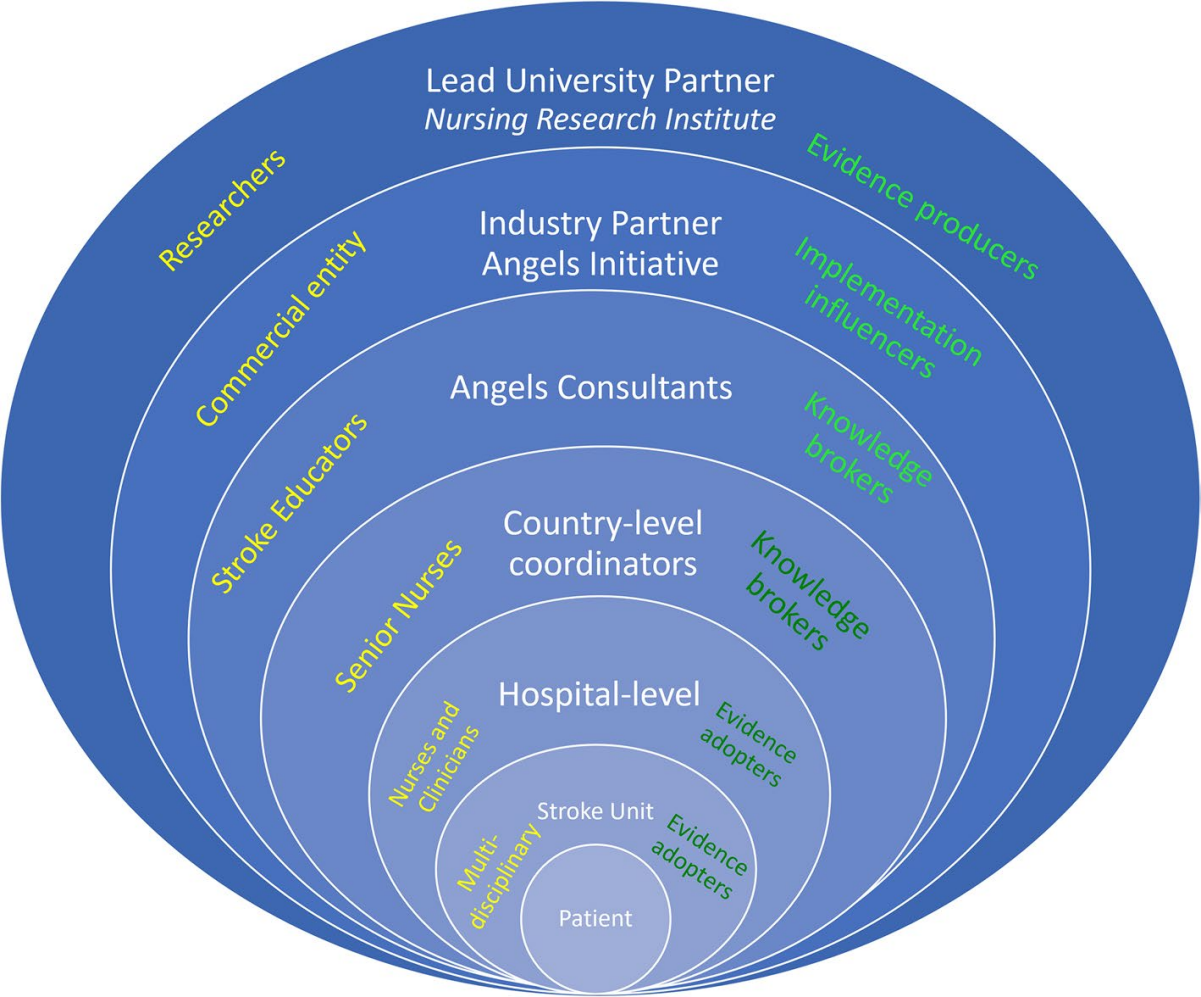
Cascading facilitation: Key stakeholder organisations and individual groups involved in implementing evidence-based clinical protocols for acute stroke care. Yellow font (left hand side) = role outside of project, name, Green font (right hand side) = project role action

 Our model of international upscale involved multiple stakeholders throughout all project phases. We describe this approach as *cascading facilitation* (Fig. 1). Similar to cascade ‘train the trainer’ models used widely in healthcare and industry for information flow from one group to another until it reaches its final destination [21–24] the flow of facilitation phases follow a similar cascading pattern. The first phase in Fig. 1 shows the involvement of key stakeholder groups (i.e., the academic researchers [evidence producers]) and not-for-profit partners (Angels Initiative [evidence translators]) to connect and initiate the collaboration. The next phase involved preparing the Angel Consultants and subsequently the Country-level Co-ordinators (Senior Nurses [knowledge brokers]), followed by preparing the multi-disciplinary hospital-based medical and nursing staff, and other stroke care personnel such as speech therapists (evidence adopters). Because of the collaboration between industry and academic partners, different expertise and experience could be combined and leveraged across all project phases to progress scale-up.

### Participants

Purposive maximum variation sampling was used to select participants for interview four different stakeholder groups (Table 1). They had to be English-speaking and (i) involved in an advisory or project management capacity (Steering Committee, Senior Project Team, Project Staff based in Australia and Europe) or (ii) hospital-facing participants (i.e., Angels Team Leaders, Consultants and nurse Country Co-ordinators) with experience of a range of hospitals at various stages of project implementation (completed, ongoing and commenced). The Angels Consultants were responsible for hospitals within one or more European countries. The aim of the maximum variation sampling approach was to capture a broad perspective from a range of participants who had different roles and responsibilities in relation to the upscale and implementation of the FeSS Protocols in a range of countries and a range of within country hospitals (project completed, ongoing and commenced). Hence clinical staff within individual hospitals were not interviewed. Potential participants were invited via email and were provided with a Participant Information Sheet, and consent form.

**Table 1:** Sampling frame for participants

Group	Stakeholders	Role
1	High level/ senior / executive and project managers and steering committee members	Responsible for QASC Europe project governance, decision-making and leadership
2	Angels Team Leaders: managers of Angels Consultants	Responsible for facilitating implementation of the FeSS Protocols into enrolled hospitals
3	Angels Consultants	Role to increase the number of patients treated in stroke ready hospitals and in relation to FeSS Protocols to facilitate and promote the use of the protocols in enrolled hospitals across a range of European hospitals. Each Angels Consultant was responsible for one or more countries.
4	Country Co-ordinators	Senior stroke nurses whose role was to promote the QASC Europe project within hospitals enrolled in their country and who also led implementation of the FeSS Protocols locally in their hospital

### Data collection

The semi-structured interview guide was drafted by a research team member (EM) and circulated to other research team members (SD, DC, SM, TF, JvdM) for feedback and suggestions. The interview guide was designed to explore participant views of factors that influenced the engagement of stakeholders, preparation for upscale and promotion of the FeSS Protocols. The interview guide included open-ended questions with prompts and probes about: (i) the participant’s role in the project; (ii) their views on whether stakeholders understood what was required of them in relation to facilitation of implementation of the Protocols and, (iii) their views on factors, including barriers and enablers, that influenced implementation. A core set of similar questions were supplemented by additional questions specific to the relevant stakeholder roles. The interview guide was pilot tested for meaningfulness and clarity in two mock interviews with members of the research team and minor revisions were made. Interviews were conducted remotely by a researcher (EM: female, PhD, Nursing, experienced qualitative health services researcher) not involved in project management using on-line video software between June and September 2020 towards the end of the pre-test/post-test study. Interviews were recorded after receiving informed written consent from the participants, and subsequently transcribed verbatim. Interviews were approximately of one-hour duration. The interviewer took notes during and after the interviews. Data saturation was reached at 22 participants. Data saturation was determined by EM and checked by the research team by assessing that the data obtained was information rich and that no new insights were evident in the data.

### Data analyses

Descriptive thematic analysis with an inductive approach was undertaken; that is, no pre-defined framework was deployed [25, 26]. Established procedures across six sequential phases were followed: all transcripts were read and re-read (phase 1 familiarise with data) with line-by-line analysis and the initial categories identified (phase 2 coding). The grouping and hierarchical consideration of related categories followed (phase 3 generating initial themes), with important themes and sub-themes subsequently refined and identified (phase 4 reviewing themes). All initial coding was completed by one coder (KB – not otherwise involved in the study in any way – female, PhD, Psychology, experienced qualitative health services researcher) using NVivo version 12 [27]. Each theme/sub-theme was subsequently reviewed by EM, SM (project director) and SD, project manager) and nuanced theme identification undertaken (phase 5 define and name themes). Preliminary themes were presented to the participant stakeholder groups for feedback to check that nothing had been missed and final themes and sub-themes were endorsed by all authors (phase 6 write up). Illustrative quotes are provided verbatim with grammar corrected.

## Results

All stakeholders approached consented to participate. Thirteen individual and three group interviews were conducted with 22 participants across the four stakeholder groups (Table 2). For the Executive/Manager/Steering Committee group, there were three representatives from each academic partners, five industry representatives (Angels Initiative), and Chair of the QASC Europe Steering Committee (external). There were three Angels Team Leaders, five Angels Consultants and five Country Co-ordinators who all had an active role in the project.

**Table 2:** Participant groups by interview type

Interview	Group	*N* = 22	Roles and scope
**Individual interviews** *n* = 13	Senior / Executive managers and Steering Committee members	9	Senior Executives or managers from Angels Initiative and NRI. Steering Committee Members included the Chair, QASC Europe Project European Liaison officer, QASC Europe Project Manager. All participants with one exception had been involved with the study since inception (2017).
	Angels Team Leaders	3	Team leaders covered countries in Central, Eastern and Western Europe. Team Leaders were in position prior to study inception.
	Angels Consultant^a^	1	With one exception, Angels Consultants had responsibility for multiple hospitals within a single European country. Angels Consultants were in position prior to study inception.
**3x Group Interviews** *n* = 9	Angels Consultants	4	
	Country Co-ordinators	3	Country co-ordinators were senior nurses who had responsibility for hospitals within a single country and were also senior stroke nurses implementing the protocols within their own hospitals. Country co-ordinators were initiated post-study inception.
	Country Co-ordinators	2	

*NRI* Nursing Research Institute, *QASC* Quality in Acute Stroke Care

^a^Scheduled as a group interview but only one participant attended. All countries had a mix of hospitals that went live or were slow to progress, with one exception where all hospitals were slow to progress

### Main themes identified

Themes and sub-themes are presented in Table 3 and are discussed below, supported by illustrative quotes. Enablers and barriers to upscale using a cascading facilitation framework are illustrated below within the identified themes. Perspectives were generally similar across all stakeholder groups.

**Table 3:** Themes and sub-themes

1. Readiness for change	2. Roles and Relationships	3. Managing multiple changes
1.1 Negotiating expectations	2.1 Defining and establishing roles	3.1 Accommodating and responding to variation
1.2 Intervention feasible and acceptable	2.2 Harnessing nurse champions	3.2 More than clinical change
1.3 Shared goal of evidence-based stroke management		3.3 Multi-layered communication framework

## Themes and sub-themes

### Readiness for change

Three sub-themes (*negotiating expectations, intervention feasible and acceptable, shared goal of evidence-based stroke management*) represent the factors that assisted with preparing for change and managing the complexity of the preparation and pre-implementation phase.

#### Negotiating expectations

Strategies were negotiated to manage different expectations about project scope, timelines and facilitation approach. While executive group participants said the Angel Consultants would ‘just do it’ (i.e. deliver the protocols to the hospital personnel in the hospitals covered by each Angels Consultant as an extended part of their current role) and support efforts to implement, the researchers brought an implementation science perspective focused on the employment of formal behaviour change strategies.

*I don’t think that we (researchers and industry) understood each other in the beginning (*Participant 2, Group 1).

*I’m coming from a scientific background where I know the evidence about clinician behaviour change and it's not as simple as just telling clinicians alone* (Participant 2, Group 1).

In addition, participants reported an expectation that the industry partner wanted an immediate start. Both of these factors required negotiation between the industry and academic partners and led to a compressed planning period.

*Projects with industry are always interesting, because they really force you to streamline things.* (Participant 1, Group 1).

*But if I did it all again, I would like to have more preparation time at the sites.* (Participant 2, Group 2)

Related to expectations of the industry partners for rapid implementation, there was discussion about the project scope and design (that is, whether QASC Europe was a research study or quality improvement initiative), and the need to collect reliable data that was important to the researchers to provide evidence of impact.

*That was a bit of a sticking point because industry stakeholders wanted to move quickly and the hospitals weren’t prepared to do their baseline [data]; “oh well, let’s just move on without it”. It’s like, no, because then you’ll never know whether it worked and we’d have nothing to say apart from 60 hospitals have started it and they say they’ve done it. *(Participant 1, Group 1)

From the Angels Consultants’ perspective, the scope of the project also impacted upon expectations about how much support they could give inexperienced hospital staff. The researchers required pre-and post- data for stroke patients (processes of care for fever, sugar and swallow monitoring and management) in order to demonstrate practice change. Many hospital clinicians were inexperienced in data collection. The database used was the Registry of Stroke Care Quality (RES-Q) that captures performance and quality measures enabling international comparison of stroke care quality. Some hospitals were already participating in this registry and others were not. Hospital clinicians were provided with generic support as needed from the Australian-based Project manager, the Angels Consultant and RES-Q support person and were also provided with educational resources and data dictionary. Preparing and supporting sites for data collection required significant input from the team and there was a sense that the project evolved into something bigger than anticipated.

*…the whole project has been a progression over time in terms of one small focus to going into a bigger focus.* (Participant 6, Group 1)

#### Intervention feasible and acceptable

An enabler was that the QASC Europe project was perceived as a worthwhile ‘high value’ international project. The protocols were regarded as feasible and acceptable for implementation across a range of hospital settings and countries with variable access to resources, given their simplicity and scientific evidence base. For some participating hospitals, implementation of the protocols was something that could be introduced to improve stroke outcomes in the absence of other emergency medical treatments for acute stroke; for example, several sites were not able to access thrombolysis. Monitoring and managing temperature, glucose and swallowing processes were reported by Country Co-ordinators as easily integrated into existing care pathways.

*Because of course the project was great you know and it’s crystal clear. You have these three protocols that you have to implement. *(Participant 10, Group 2)

*… [the] QASC project shows that … if we just measure and control three measurements … it could change the life of patients. *(Participant 11, Group 3)

#### Shared goal of evidence-based stroke management

All stakeholders shared the goal of improving stroke care and this was an overarching enabler to positive outcomes from negotiations between industry and academic partners and also for the implementation process. Other goals shared by stakeholders was to raise awareness amongst hospitals about the benefits to patients from evidence-based stroke care beyond pharmaceuticals or devices and promoting nurse-led evidence translation in European countries.

*This was great for the nurses, you have all the literature that is supporting the credibility of the project. *(Participant 8, Group 1)

These shared goals engendered high levels of engagement between collaborators that in turn led to high commitment in terms of investing time and resources to progress the project:

*The importance of the QASC Project … it was very good … was when we went to the hospital, we had something more to propose, something very relevant for the hospitals … so the FeSS tools were … very useful for the hospitals.* (Participant 10, Group 2)

### Roles and relationships

The two subthemes *defining and establishing roles* and *harnessing nurse champions* reflect that the supportive ethos across the collaboration was an enabler. However, expectations and understanding of stakeholder roles required ongoing negotiation and refinement. Being responsive to the need for additional roles not originally conceptualised was necessary. This included the creation of an Angels Initiative QASC Team Leader and Country Co-ordinators to support implementation and to strengthen the cascading facilitation framework.

#### Defining and establishing roles

Initially there was not a clear role definition or expectations in relation to Angels Consultants’ role and responsibilities. The project was one of many responsibilities for the Angels Consultants and added to an existing workload.

*… at the beginning they had difficulties to understand what was their role, what they were supposed to do. QASC was not their priority, and not included in their workplan* (Participant 10, Group 2).

Although the Angels Consultants were identified as the link between the research team and the hospital staff, prior to involvement in QASC Europe, their standard role within the hospitals was to work with emergency department clinicians. Their role was to work with the hospitals to become ‘stroke ready’, assisting to identify gaps in evidence-based treatments for the hyper-acute stage of stroke management. Having to liaise with stroke unit clinicians in the stroke units where the FeSS Protocols would be implemented was an extension to their existing role.

*Because of course they were super comfortable with working in the hyper-acute phase and working with the emergency physicians and doing the simulations* (Participant 12, Group 1).

The impetus to commence rapid implementation coupled with their additional workload led to delayed site initiation by Angels Consultants at some hospitals. Enablers identified within this theme included the establishment of additional roles to support the Angels Consultants in taking on the additional workload. A *QASC-specific Angels Team Leader role* was filled by an Angel Consultant who had successfully worked with hospitals to complete all the requirements of QASC Europe and, as such, was a valuable resource and QASC Europe mentor for all Angels Consultants. The establishment of this role helped to integrate the QASC Europe project as part of the Angels Consultants workload.

*I think that in the beginning I have so many other things to do and now it’s coming, this QASC, and it was like a lot of collision. But now the project is really part of process once it formally got integrated into our workload and we have this national co-ordinator who is part of the Angels’ Initiative.* (Participant 11, Group 3)

A number of other roles were fundamental to the success of implementation including the Australian-based Project Manager. Their extensive experience as the original QASC trial (and related QASC studies) Program Manager was viewed as a significant enabler to the remote initiation and facilitation of the European roll-out.

*They have to be absolutely, completely across this in their sleep because there’ll be stuff that will come. They’ll need to be able to have the gravitas and the knowledge to make decisions.* (Participant 2, Group 1)

A European-based Project Liaison role established prior to project commencement. was anticipated to be instrumental for ensuring local, real-time support and troubleshooting for the Angels Consultants. Both roles helped to support the Angel consultants to understand the project goals, implementation science principles and research processes.

*… the European Project Liaison officer was pivotal… they were texting her and …there was instant real time support* (Participant 8, Group 1).

Over the course of the project, a need emerged for Country Co-ordinators who were senior stroke nurses who could provide a central point of contact for all participating hospitals within their countries. These ‘early adopters’ who had successfully implemented the protocols in their own hospitals then supported and mentored their colleagues at other hospitals. Although based in different organisations, the Angels Consultants and the Country Co-ordinators worked together and shared knowledge each providing their own experience with QASC Europe and role expertise to support clinicians to facilitate protocol implementation.

*We jointly identified that training the nurses on this post-acute phase was lacking in most of our hospitals; not only about the importance of monitoring these parameters and the importance to the patient, but also the importance of collecting data and how to implement a study like this* (Participant 2, Group 4).

#### Harnessing nurse champions

The project had a specific impact on nursing in participating hospitals and this was one of the most significant benefits of, and enablers for the project. Facilitation of FeSS Protocols into each hospital was led by a stroke nurse. For many, this was the first time that a nurse, rather than medical staff, had led hospital practice change or been involved in a research project/ quality improvement initiative.

*In a lot of countries, the nurses are not involved in this kind of study. It is more the physicians, but this is changing. This role of the nurses is changing.* (Participant 12, Group 1)

Country Co-ordinators reported that the project provided an example on how to implement evidence-based change in local settings and on quality monitoring processes as well as giving nurses exposure to behaviour change principles and strategies.

*I think that it’s a really empowering project. You really see, especially when you’re working with nurses, you really get huge feedback. This is a very big reward to see how much they appreciate you bringing this project that is just the beginning for them.* (Participant 14, Group 3)

Despite having to manage multi-level changes, relationships, and processes to achieve protocol implementation, there was high engagement with the project by nurses. Participation in the project was perceived as giving nurses ‘visibility,’ elevated their status as a practice change expert and highlighted how care for patients with stroke can be improved by nurse-led protocols. Some Country Co-ordinators reported that they had subsequently been asked to assist with other evidence translation projects on the basis of their QASC Europe experience, thus the benefit of participating extended beyond QASC Europe.

*The most important thing of the FeSS Protocol and the QASC study is that it gives importance to the nurses. So, these are the things that were really very well received by the hospitals. I didn’t see any barrier or any issue from their side to implement the FeSS Protocol.* (Participant 15, Group 3)

### Managing multiple changes

The capacity for all levels of the cascading facilitation framework to manage multiple changes involving numerous stakeholders, accommodate within and between country variation and evolve a communication framework was essential for optimal scale-up. This is reflected in the sub-themes of *accommodating and responding to variation, more than clinical change* and *multi-layered communication framework.*

#### Accommodating and responding to variation

The ability and capacity to manage multiple changes in processes within existing workloads to progress the project across diverse country and hospital settings with variable experience of implementing evidence-based care, was an enabler. Wide variation existed between and within countries and hospitals. For example data collection, access to resources such as computers or blood glucose monitors, different staffing models and experience in facilitation of evidence translation. This required, from those such as Angel Consultants and Country Co-ordinators who worked across different countries and hospitals, a flexibility and ability to understand, be responsive to and to accommodate variation:

*Need to understand and acknowledge country differences. You can’t just transfer processes that work in one country to all countries.* (Participant 3, Group 1)

An additional issue was the responsibility for multiple hospitals by Angels Consultants in diverse countries with different health systems. Based on their prior knowledge of a country’s health system or hospital, Angels Consultants and Country Co-ordinators had in-depth knowledge and tailored strategies to engage and support hospitals.

*It was [a] very different approach and [a] very individual and tailored approach for engaging all [of] the hospitals*. (Participant 8 Group 1)

The wide-ranging differences in role and autonomy of nurses across countries required additional support from Angels Consultants and Country Co-ordinators in order to facilitate protocol implementation. Different intensities of involvement in terms of promoting the protocols and providing facilitation advice were required from the Consultants and Country Co-ordinators, depending on how experienced the individuals and hospitals were in evidence translation:

*It’s been such an eye-opener seeing how nursing works in other countries and the fact that they don’t have a similar level of autonomy.* (Participant 3, Group 1)

This meant that for some sites, the Angels, Country Co-ordinators and on the ground project staff such as the European Liaison Officer, had to devote more time than anticipated to some sites to overcome barriers related to variation.

#### More than clinical change

While the aim of the QASC project was clinically focused, that is to implement the FeSS Protocols in hospitals, other changes were required to enable protocol uptake at different levels across participant groups. These changes were largely linked to expanding roles to enable promotion and facilitation of changes at the hospital level.

As previously mentioned, the role of the Angels Consultants had to expand to incorporate post-acute patient care within the stroke unit. This was a significant change for the Angels Consultants, despite having working relationships in place at hospitals.

A second major change was that in many hospitals, medicine was seen as the discipline primarily responsible for changing clinical practice, hence these hospitals had little or no experience in nurse-led evidence translation and required education about nurse-led practice change.

*It’s been more a project of learnings in behavioural science than it’s been in stroke care I’d say …* (Participant 2, Group 1)

In addition, some protocol elements required doctor involvement (e.g., prescribing insulin therapy), hence this required that the nurse’s role extended to coaching and prompting doctors in the use of the protocols. This shift in roles from medical to nurse leadership of clinical change and evidence translation posed a significant cultural change that had to be overcome. Responsibility for this largely rested with the nurses supported by the Angels Consultants and Country Co-ordinators:

*I had to change the logic of doctors. That was really problematic … I think because… first time ever a nurse changed something* (Participant 16, Group 4).

Therefore another level of change that was required was that the Angels and Country Co-ordinators had to negotiate and engage with hospitals and medical staff to ‘sell’ the value of clinical nurse leadership to initiate the protocols across the multidisciplinary team, and this required additional effort:

*Of course, there are barriers in the hospital administration, or the head of the departments, and they might not see immediately the value of initiatives like this. So you have to push it a little bit. I think it depends on the countries* (Participant 15, Group 4)

From the researchers’ perspective, facilitating and monitoring multiple levels of change was required. For example, negotiating the addition of the project to the existing workload of the Angels Consultants; providing education to the Angels Consultants and Team Leaders about clinician behaviour change; monitoring the efforts of the Angels Consultants to promote use of the protocols; also supporting Country Co-ordinators to negotiate with hospitals to enable acceptance of nurse-led protocols:

*So, we had to change their [Angels] behaviour and help them understand how they could sell it to the clinicians.* (Participant 2, Group 1).

#### Multi-layered communication framework

Formal communication and a support framework were pivotal in addressing some challenges. Given the different groups of stakeholders, it was essential to prevent communication lapses or miscommunication. The geographical range, number of countries and hospitals meant that many stakeholders, including those in project roles, had to work remotely with limitations such as time differences.

*I remember asking in the very beginning, do we have [a] deadline … [to implement] and there was not a clear answer.* (Participant 16, Group 4)

The multi-modal communication used within the project, across stakeholders was perceived as an important enabler. Various communication tools and systems were used, as relevant to project role and phase, supporting communication. Adapting *communication as relevant for different stakeholders* proved an important aspect of implementation. A centralised, formal system was augmented with real-time text messaging by the European based project personnel to the European Liaison Officer to ensure queries were answered promptly. As previously mentioned, a *QASC-specific Angels Team Leader role* was linked to an existing role who provided a pivotal central point of contact for all Angels Consultants to streamline the communication.

*So, I think this is a really, really good learning to have this role for other countries, especially if you are gathering a group of countries that they don’t have English as their mother tongue.* (Participant 14, Group 3)

## Discussion

Our aim was to identify factors that influenced the upscale of the FeSS Protocols into multiple European hospitals as part of the international scale-up [15], of the successful Australian-based QASC trial [2]. Our multi-stakeholder industry-academic collaboration used a scaling-up strategy that we call *cascading facilitation*, with nurses at the country and hospital-level driving change with external support as shown in Fig. 1. This framework may provide an alternative approach to other scale-up frameworks, which do not show working relationships between stakeholder organisations [8, 28]. 

While industry-academic collaborations are encouraged by government funding bodies in many countries in order to achieve research impact [29], our results highlight the complexity and advantages of global up-scaling via multi-stakeholder/university-industry collaborations. Although there was agreement on the shared aim and the role that the collaboration could play to improve stroke care, establishing detailed understanding on the steps required for the project to be actioned for all stakeholders took time and required constant negotiation. The importance of time and flexibility for such negotiations has also been identified in previous research as necessary for both multiple stakeholder health services research [29] and for university-industry collaborations [9, 10]. 

In addition, the expectations and understanding for each stakeholder group initially required clarification. Differences in approaches and operational timescales between industry partners and researchers have been documented in other research as requiring addressing at the outset [30]. For example, for Angels Consultants, the focus on post-acute hospital care was outside of their usual scope of practice and experience which previously had been focused on the hyper-acute phase of stroke and hence, facilitation of the new FeSS Protocols were perceived as additional to the Angel Consultants’ role and not core business. It therefore took time for ownership, a key attribute of change agents to occur [16, 31]. However, a shared aim of improving stroke care in hospitals and that the project was seen as high value was a powerful enabler for addressing issues that arose.

Willingness and flexibility to undertake new activities within existing roles, including Angels Consultants’ workflow across the full stroke journey and nurses’ leading evidence-based change, was fundamental to the scale-up. Such flexibility has been identified as an important enabler for university-industry collaborations and evidence translation scale-ups [9, 16, 20]. Recent work has illustrated that organisations can vary in how they adapt to change (i.e., incorporators, early investors or learners) and the various paths that may be effective to sustaining change [32]. Additional barriers such as country-level variation across settings and hospital-level variation, was also a factor that needed more attention than initially expected.

Three different stakeholder groups have previously been identified as important for scaling up [33] (Fig. 1): *evidence producers* (academic partner) – test innovation, present findings; *evidence influencers* (industry partner) – determine whether to facilitate scale-up; and *evidence adopters* (hospital setting) – decide whether to adopt or not. Using cascading facilitation required linking the outer evidence producers (academic partner, QASC/FeSS Protocols) and inner evidence adopters (hospital setting, stroke unit) contexts. That is, a connecting role was required, in this case the Angels’ Initiative consultants provided the connecting role [34]. These roles are variably referred to as boundary spanners [35], brokers [36] or bridges [37] but also have been identified as purveyors/intermediaries (as a bridging factor); a new factor in the EPIS (Exploration, Preparation, Implementation, Sustainment) framework [38]. This role can be held by an organisation or an individual, and can provide support, consultation or and/training [38]. Importantly, they do not influence the evidence per se, but influence where or how the evidence can be implemented. This role was critical to the success of the upscale of the protocols.

In our cascading facilitation framework, there were multiple roles performing as knowledge brokers; that is, not only providing a link, but also identifying and addressing jointly issues that arose. For example, site-specific issues; identifying, interpreting, and translating research evidence for local practice. Angel Consultants (and their Team Leaders) and the Country Co-ordinators were not only acting as knowledge brokers, but also connecting different stakeholder groups while building capacity in nurse-led practice change. Addressing capacity constraints has previously been identified as a factor important for university-industry collaborations [9]. In addition to individual attributes such as interpersonal, communication and motivation skills [33, 36], knowledge brokers need expertise about the intervention (the outer academic partner domain) and the end user (the inner, hospital setting) to be successful [36]. Having boundary spanners/knowledge brokers already connected with and known to specific hospitals provided an important advantage for our collaboration.

Several positive outcomes and benefits from the QASC rollout in Europe were identified. Some of these are similar to those previously identified for university-industry collaborations [9]. For the academic partners, the project provided the opportunity for expansion of the FeSS Protocols into 64 hospitals; development of a set of standardised multi-lingual documentation for future roll-outs and the evaluation of the cascading facilitation framework as a basis for future university-industry collaborations. The project benefited from being able to capitalise on the industry partner’s established Angels Initiative network and well-established processes to achieve behaviour change in European hospitals. The Angels’ pre-existing relationships within hospitals was key for scale up and reach. Our findings suggest the importance for others to identify similar personnel with established relationships through professional networks and communities of practice that can be leveraged for reciprocal benefits [38–40]. 

For the industry partner, participating in QASC Europe extended the reach of the Angels Initiative beyond the hyper-acute phase to the acute care phase and increased knowledge and awareness of implementation science. In the hospital setting, positive outcomes included raising awareness of optimal stroke care and hospital workforce capacity for clinical care and inter-disciplinary working relationships benefited from a raised awareness of nurse-led evidence implementation and of having nurses at the forefront of practice change. The results of the main pre-test/post-test study showed statistically significant improvements in recording of measurements for all three FeSS components, [15] and this suggests that QASC Europe has established that nurse-led evidence translation projects are accepted and influential in hospital settings, even those where the nurse traditionally has not had a role in leading change.

This study has a number of strengths including an *a priori* contemporaneous examination of scale-up at an international level for evidence-based protocols, from the initial researchers to multi-stakeholder representation from all relevant groups across multiple countries able to reflect on the project from inception. Angels Team Leaders, Angels Consultants and Country Co-ordinators were able to speak to experience across multiple hospitals. The qualitative approach enabled rich data to draw on, providing detailed barriers and enablers to scale-up using cascading facilitation. In addition, the researchers were not aware of the results of thepre-test/post/test study conducted in Europe.

A number of limitations also need to be considered. One was that research team were English-speaking, and thus interviews were conducted in English. We also did not interview clinicians who were tasked with delivering care according to the protocols as the aim was focused on country-level, rather than hospital-level experience and on the factors leading up to hospital level engagement and readiness focusing on the university-industry collaboration. The impact of COVID-19 is unknown: the interviews were conducted remotely (rather than as planned face-to-face during a European stroke conference), and implementation occurred during the pandemic, limiting face-to-face interactions across stakeholder groups and individuals.

## Conclusions

A cascading facilitation collaborative model of evidence translation achieved implementation scale-up at pace across a wide geographical area in multiple hospitals. Despite differences in perspectives and foci, implementing evidence-based clinical change within hospitals can be initiated outside of the healthcare system and facilitated by collaborative university-industry partnerships with the shared goal of providing optimal care for patients. Cascading facilitation may benefit future international evidence translation scale-ups.

### Supplementary Information

**Additional file 1.** Interview Guide: QASC Europe Process evaluation.

**Additional file 2.** QASC Europe Steering committee. QASC Europe Study Implementation Committee.

## Data Availability

The datasets used and analysed during the current study are available from the corresponding author on reasonable request.
