# Supplementary material for: The Quality in Acute Stroke Care (QASC) global scale-up using a cascading facilitation framework: a qualitative process evaluation
Source: BMC Health Serv Res. 2024 Jan 29;24:144. doi: 10.1186/s12913-024-10617-9 (PMC10823736; doi:10.1186/s12913-024-10617-9)
Supplement: Supplementary file 1 — Additional file 1. Interview Guide: QASC Europe Process evaluation. [file 12913_2024_10617_MOESM1_ESM.pdf]

Interview Guide: QASC Europe Process evaluation

HIGH LEVEL MANAGERS

Aims:

To identify views and perspectives on processes and success factors relating to the QASC Europe project.

Preamble

Thank you for agreeing to speak with me today. My name is Professor Elizabeth McInnes and I am conducting interviews with selected groups of people who were involved in various ways in the implementation of the FeSS protocols for the QASC Europe project.

These interviews will give us valuable information on your views about what worked well what didn't work as well and your suggestions for increasing uptake.

You have been selected for this interview because of your role as *(insert role here)* and that you are able to give an informed perspective about your experiences in the QAS Europe project.

If you are agreeable, I will take notes and record the interview so that I don't miss any details and can carry on an attentive conversation with you. All interviews are confidential and anonymised and you (and your hospital) will not be identified in any publication.

Do you have any questions?

<u>Consent form instructions</u>

Check that the interviewee has read the participant information sheet and read and signed consent forms. They keep a copy and the researcher keeps a copy as well.

Focus group Country Co-ordinators Guide: QASC Europe Process evaluation

**Aims:**<br>To identify views and perspectives on the factors that influenced the introduction, implementation and uptake of the FeSS clinical protocols into practice.

Preamble
Thank you for agreeing to speak with me today. My name is Professor Elizabeth McInnes and I am conducting interviews with selected groups of people who were involved in various ways in the implementation of the FeSS protocols for the QASC Europe project.

These interviews will give us valuable information on your views about what worked well what didn't work as well and your suggestions for increasing uptake.

You have been selected for this interview because of your role as *(insert role here)* and that you are able to give an informed perspective about your experiences in the implementation of the protocols.

If you are agreeable, I will take notes and record the interview so that I don't miss any details and can carry on an attentive conversation with you. All interviews are confidential and anonymised and you (and your hospital) will not be identified in any publication.

Do you have any questions?

Consent form instructions
Check that the interviewee has read the participant information sheet and read and signed consent forms. They keep a copy and the researcher keeps a copy as well.

PART A

2. Level of support provided

2.1 What did your role in QASC Europe entail?

2.2 What was your understanding of your role in relation to QASC Europe?
PROBE: Did they feel adequately prepared/trained for the role?

2.3 Did you feel adequately prepared and supported and what were your main sources of support?

2.4 What was needed to support you in this role that was not available?

3. Implementation of FeSS Protocols

3.1 Do you think hospital staff understood what was needed in relation to implementing the FeSS protocols into their daily practice?
PROBE:
If 'yes' what helped?
If 'no' what hindered?

3.2 What do you consider were the key factors that assisted hospitals to implement the protocols?

PROBES	RESPONSES
1. Internal and external staff<br>*Who?*	
2. Training and education<br>*Adequacy?*	
3. Designated internal facilitator/champion<br>*Who?*	
4. Resources<br>*What?*	
5. Engagement of multidisciplinary team?	
6. Other	
7. Other	
8.Other	

3.3 What was your role in increasing awareness of and uptake of the protocols in the hospital?

PROBE:
What were the things that you did to help to promote implementation and uptake?

What other things were done that you are aware of that promoted implementation and uptake?

Did you use any of the resources – if so which ones?

3.5 Which clinical protocol do you think were the easiest and most difficult to incorporate into practice?
Prompt: Fever, sugar, swallow (FeSS) protocols.
Probe: Reasons for ease or difficulties; Of those that were difficult how did they address these difficulties

3.6 What do you think were the internal and external factors that had a positive or negative impact upon the implementation of the protocols?
Probe: Changes in staffing, introduction of a new policy (either Government, hospital level), new guidelines, resources, other research study, competing demands etc

3.8 How useful were the resources provided? (*ie: protocols, implementation strategies, videos, barriers and enablers worksheets*)

3.9 What else was needed to introduce the protocols into practice and to change practice?

4. Barriers and facilitators to implementation *(NB: see table below and fill)*
From your point of view:
4.1 What are/ were the main barriers to implementing the FeSS protocols?

4.2 What helped or would help to overcome these barriers?
Prompt: staff, organisation, resources, local policies, organisational culture, competing priorities.

4.3 In terms of these barriers, did they require minimal, moderate or significant input from yourself to prevent them from impeding implementation?

4.4 What are the things that you think were most needed that were not available to promote uptake?

**Main barriers to implementing FESS protocols**	**What helped to overcome them?**	**What would have helped to overcome them?**	**To address these barriers was minimal, moderate or significant input from you required?**	**What were the things that you think were most needed that were not available to promote uptake?**

5. Sustainability

5.1 What do you think will help to enable sustainability of the protocols into routine practice?

6. Other questions

6.1 What do you feel have been the main benefits/outcomes of involvement in QASC Europe? To you as an individual, the Angels initiative, the hospital?

6.2 What recommendations do you have for other individuals and hospitals implementing the protocols in the future?

6.3 Do you think the QASC Europe project will inform future implementation work? \Have you seen any evidence of this already occurring in any hospitals?

6.4 Did you see any growth in nurses role/ autonomy in terms of an increased effort or focus to drive quality stroke care process during the term of the QASC Europe Project?

7. Final thoughts and comments

7.1 Is there anything else that you would like to add?

Thank you for your valuable time and input

PART A: Interviewee characteristics

1.1 QASC Europe role designation ______________________________________

High level project management ☐

Angel team leader ☐

Angel consultant ☐

Country Co-ordinator ☐

1.2 Length of time in current role (months/years) _____________________________

PART B: Interview questions

1. What did your role in relation to QASC Europe entail?

2. What was it about the research that captured your interest?

3. Have you been involved in similar translational initiatives?

4. Tell me about the efforts in relation to preparation and planning of how QASC Europe would be implemented?

5. Were expectations clear about communication, timelines, roles and responsibilities?

6. Do you think that all key parties, eg NRI, Angels T/L and consultants and clinicians, understood what was needed to make QASC Europe happen?

7. How were stakeholder interrelationships and communication strategies (between the various parties, Angels, hospital staff) managed?

8. What insights have you had about clinician behaviour change as a result of being involved in QASC Europe?

   Is there anything that can be transferred to other projects in the future?

9. What do you feel have been the main benefits/outcomes of QASC Europe? How has it value added to the work of your organisation and/or to individuals?

10. What was done well and what could have been done better?

a. *If you did it again, what would you do differently?*

b. *What were the difficulties?*

c. *Internal and external factors that had a positive or negative impact upon project success, eg. communication; resources; training; expectations.*

d. *Minimal, moderate significant input required to solve*

11. Do you have any insights on the basis of QASC Europe about translational research involving industry and University research institutes?

    a. Would you do it again?

    b. Was it worth it or too much trouble?

    c. What will be the legacy of QASC Europe from your point of view?

12. What recommendations do you have for others interested in similar translational projects in the future?

13. Is there anything else that you would like to add?

Thank you for your valuable time and input

Focus group and Interview Guide

QASC Europe Process evaluation – ANGELS CONSULTANTS

**Aims:**<br>To identify views and perspectives on the factors that influenced the introduction, implementation and uptake of the FeSS clinical protocols into practice.

Preamble

- Thank you for agreeing to speak with me today. My name is Professor Elizabeth McInnes and I am conducting interviews with selected groups of people who were involved in various ways in the facilitation or implementation of the FeSS protocols for the QASC Europe project.

- These interviews will give us valuable information on your views about what worked well what didn't work as well and your suggestions for increasing uptake.

- You have been selected for this interview because of your role as *(insert role here)* and that you are able to give an informed perspective about your experiences in the implementation of the protocols.

- If you are agreeable, I will take notes and record the interview so that I don't miss any details and can carry on an attentive conversation with you. All interviews are confidential and anonymised and you (and your hospital) will not be identified in any publication.

- Do you have any questions?

Consent form instructions

Check that the interviewee has read the participant information sheet and read and signed consent forms. They keep a copy and the researcher keeps a copy as well.

PART A

1. Interviewee characteristics

1.1 QASC Europe role designation ____

High level project management ☐

Angel team leader ☐

Angel consultant ☐

Country Co-ordinator ☐#

#

1.2 Length of time in current role (months/years) ______________________________

1.3 Professional background, years of experience, brief details of role (tick all that apply):

a. Clinical ☐
Number of years ______
Brief details of role _______________

b. Stroke specific ☐
Number of years ______
Brief details of role _______________

c. Research ☐#
Number of years ______
Brief details of role _______________

d. Evidence translation / practice change ☐#
Number of years ______
Brief details of role _______________

e. Quality improvement ☐#
Number of years ______
Brief details of role _______________

f. Other ☐_________
Number of years ______
Brief details of role_______________

1.4 Previous involvement in practice improvement or evidence translation - role and where?

____

____

____

____

1.5 List countries that participant is involved

____

____

____

____

PART B

2. Level of support provided

2.1 What did your role in QASC Europe entail?

2.2 What was your understanding of your role in relation to QASC Europe?
PROBE: Did they feel adequately prepared/trained for the role?

2.3 Did you feel adequately prepared and supported and what were your main sources of support?

2.4 What was needed to support you in this role that was not available?

3. Implementation of FeSS Protocols

3.1 How easy or difficult did you find your role in terms of talking with clinicians about practice change and not actually being a clinician yourself.

3.2 Do you think hospital staff understood what was needed in relation to implementing the FeSS protocols into their daily practice?
PROBE:
If 'yes' what helped?
If 'no' what hindered?

3.3 What do you consider were the key factors that assisted hospitals to implement the protocols?

PROBES	RESPONSES
1. Internal and external staff<br>*Who?*	
2. Training and education<br>*Adequacy?*	
3. Designated internal facilitator/champion<br>*Who?*	
4. Resources<br>*What?*	
5. Engagement of multidisciplinary team?	
6. Other	
7. Other	
8.Other	

3.4 Which clinical protocol do you think the nurses / doctors found the most difficult to incorporate into clinical practice?
Prompt: Fever, sugar, swallow (FeSS) protocols.

Probe: Reasons for ease or difficulties; Of those that were difficult how did they address these difficulties

3.5 How useful were the resources provided? (*ie: protocols, implementation strategies, videos, barriers and enablers worksheets*)

4. Barriers and facilitators to implementation *(NB: see table below and fill)*

From your point of view ie from YOUR ROLE:

4.1 What are/ were the main barriers that you encountered in your role in relation to QASC Europe?

4.2 What helped or would help to overcome these barriers?
Prompt: staff, organisation, resources, local policies, organisational culture, competing priorities.

4.3 In terms of these barriers, did they require minimal, moderate or significant input from yourself to prevent them from impeding implementation?

4.4 What are the things that you think were most needed that were not available to promote uptake?

**Main barriers to implementing FESS protocols**	**What helped to overcome them?**	**What would have helped to overcome them?**	**To address these barriers was minimal, moderate or significant input from you required?**	**What were the things that you think were most needed that were not available to promote uptake?**

5. Sustainability

5.1 What do you think will help to enable sustainability of the QASC Europe project?

6. Other questions

6.1 What do you feel have been the main benefits/outcomes of involvement in QASC Europe? To you as an individual, the Angels initiative, the hospital?

6.2 Do you think the QASC Europe project has or will provide nurses/doctors with any tools or techniques that could be used on other quality projects (stroke or other) ? Have you seen any evidence of this already occurring in any hospitals?

Prompt: an understanding of QI processes, how to do audit, how to use audit results to drive practice change, working on other QI activities

6.3 Did you see any development in the role of the nurse in relation to driving quality stroke care process?

6.4 What recommendations do you have for other individuals and hospitals implementing the protocols in the future?

7. Final thoughts and comments

7.1 Is there anything else that you would like to add?

Thank you for your valuable time and input
