# Supplementary material for: The Quality in Acute Stroke Care (QASC) global scale-up using a cascading facilitation framework: a qualitative process evaluation
Source: BMC Health Serv Res. 2024 Jan 29;24:144. doi: 10.1186/s12913-024-10617-9 (PMC10823736; doi:10.1186/s12913-024-10617-9)
Supplement: Supplementary file 2 — Additional file 2. QASC Europe Steering committee. QASC Europe Study Implementation Committee. [file 12913_2024_10617_MOESM2_ESM.docx]

**QASC Europe Steering committee**

Pfeilschifter W (Chair)^4&5^, Cadilhac DA^6&7^, Casertano L^8^, Cassier-Woidasky AK^9^, Cheung NW^10^, Crivorucica I^11^, Dale S^1&2^, D’Este C^12&13^, Ezoyen O^14^, Glahn J^15^, Grecu A^16^, Grimshaw JM^3^, Jarmak O^17^, Levi C^18&19^, Koláčná T^20^, McInnes E^1&2^, Medukhanova S^21^, Messchendorp G^22^, Middleton S^1&2^, Quinn C^23^, Rijksen M^24^, Salvat Plana M^25^, Salselas S^26^, Sanjuan Menendez E^27^, Skrzypek-Czerko M^28^, & Urso A^29^.

^1^ Nursing Research Institute, St Vincent’s Health Network Sydney, St Vincent’s Hospital Melbourne; and Australian Catholic University, Sydney, Australia

^2^School of Nursing, Midwifery and Paramedicine, Australian Catholic University, Sydney, Australia

^3^ Ottawa Health Research Institute, Ottawa Hospital - General Campus, Centre for Practice-Changing Research (CPCR); and University of Ottawa, Ottawa, Ontario, Canada

^4^ Department of Neurology and Clinical Neurophysiology, Städtisches Klinikum Lüneburg, Lüneburg, Germany

^5^ Department of Neurology, Goethe University, Frankfurt am Main, Germany Centre of Neurology and Neurosurgery, University Hospital Frankfurt, Frankfurt, Germany

^6^ Translational Public Health Division, Stroke and Ageing Research, School of Clinical Sciences, Monash University, Melbourne, Australia

^7^ Public Health, Stroke Division, The Florey Institute of Neuroscience and Mental Health, University of Melbourne, Melbourne, Australia

^8^ Hospital Network Planning, Lazio region, Italy

^9^ Saarland University of Applied Sciences, Saarbrücken, Germany

^10^ Centre for Diabetes and Endocrinology Research, Westmead Hospital, University of Sydney, Sydney, NSW, Australia

^11^ Institute of Emergency Medicine, Moldova

^12^ National Centre for Epidemiology and Population Health (NCEPH), Australian National University, Canberra, Australia

^13^ Sax Institute, Sydney, Australia

^14^ Erebouni Medical Centre, Armenia)

^15^ Department of Neurology Johannes Wesling Klinikum, Germany)

^16^ International Clinical Research Centre, Neurology Department, St. Ann’s University Hospital and Masaryk University, Brno, Czech Republic

^17^ Kharkiv Railway Clinical Hospital, Ukraine)

^18^ John Hunter Health and Innovation Precinct, HNE LHD, New Lambton, NSW, Australia

^19^ Department of Medicine, University of Newcastle, Callaghan, NSW, Australia

^20^ Fakultní nemocnice Motol, Prague, Czech Republic

^21^ National Center for Neurosurgery, Nur-Sultan, Kazakhstan

^22^ University Medical Center Groningen, Groningen, Netherlands

^23^ Prince of Wales Hospital, Randwick, Australia

^24^ SPM Consult, Netherlands

^25^ Health Department, Agency for Health Quality and Assessment (AQuAS), CIBER Epidemiología y Salud Pública, CIBERESP, Stroke Programme,Barcelona, Spain

^26^ ULSNE Macedo de Cavaleiros, Macedo de Cavaleiros, Portugal

^27^ Vall d’Hebron Hospital Universitari, Barcelona, Spain

^28^ Medical University of Gdańsk, Poland

^29^ Hospital Network Area-Regional Health Department, Regione Lazio, Italy

**QASC Europe Study Implementation Committee**

Middleton S^1&2^, Dale S^1&2^, McInnes E^1&2^, Cadilhac DA^6&7^, Rijksen M^23^, Fischer T^30^, Van der Merwe J^30^, Rodrigues R^30^, & Havalda R^31^.

^1^ Nursing Research Institute, St Vincent’s Health Network Sydney, St Vincent’s Hospital Melbourne; and Australian Catholic University, Sydney, Australia

^2^School of Nursing, Midwifery and Paramedicine, Australian Catholic University, Sydney, Australia

^6^ Translational Public Health Division, Stroke and Ageing Research, School of Clinical Sciences, Monash University, Melbourne, Australia

^23^ Prince of Wales Hospital, Randwick, Australia

^30^ Head of the Global Angel Initiative, Germany)

^31^ Angels Initiative Europe, Czech Republic
